# Observations of Buried Lake Drainage on the Antarctic Ice Sheet

**DOI:** 10.1029/2020GL087970

**Published:** 2020-07-31

**Authors:** D. Dunmire, J. T. M. Lenaerts, A. F. Banwell, N. Wever, J. Shragge, S. Lhermitte, R. Drews, F. Pattyn, J. S. S. Hansen, I. C. Willis, J. Miller, E. Keenan

**Affiliations:** ^1^ Department of Atmospheric and Oceanic Sciences University of Colorado Boulder Boulder CO USA; ^2^ Cooperative Institute for Research in Environmental Sciences (CIRES) Boulder CO USA; ^3^ Scott Polar Research Institute (SPRI) University of Cambridge Cambridge UK; ^4^ Geophysics Department Colorado School of Mines Golden CO USA; ^5^ Department of Geoscience and Remote Sensing Delft University of Technology Delft Netherlands; ^6^ Department of Geology and Geodynamics Universität Tübingen Tübingen Germany; ^7^ Laboratoire de Glaciologie Université Libre de Bruxelles Brussels Belgium; ^8^ Department of Geological Sciences University of Colorado Boulder Boulder CO USA

**Keywords:** Antartica, meltwater, glaciology, hydrology, GPR, hydrofracture

## Abstract

Between 1992 and 2017, the Antarctic Ice Sheet (AIS) lost ice equivalent to 7.6 ± 3.9 mm of sea level rise. AIS mass loss is mitigated by ice shelves that provide a buttress by regulating ice flow from tributary glaciers. However, ice‐shelf stability is threatened by meltwater ponding, which may initiate, or reactivate preexisting, fractures, currently poorly understood processes. Here, through ground penetrating radar (GPR) analysis over a buried lake in the grounding zone of an East Antarctic ice shelf, we present the first field observations of a lake drainage event in Antarctica via vertical fractures. Concurrent with the lake drainage event, we observe a decrease in surface elevation and an increase in Sentinel‐1 backscatter. Finally, we suggest that fractures that are initiated or reactivated by lake drainage events in a grounding zone will propagate with ice flow onto the ice shelf itself, where they may have implications for its stability.

## Key Points

- We present the first field observations of the rapid drainage of a buried lake via hydrofracture in Antarctica
- Lake‐bed fractures are detected from ground penetrating radar prior to drainage and suggest the lake may have been preconditioned to drain
- Remote sensing analysis of digital elevation model and Sentinel‐1 microwave image differences provide additional lake evolution information

## 1 Introduction

Meltwater plays an important role in mass loss for the Greenland (GrIS) and Antarctic (AIS) ice sheets. GrIS mass loss is primarily driven by surface runoff (Enderlin et al., 2014; van den Broeke et al., 2009), while on the AIS, meltwater that ponds on the ice shelves surrounding the continent may indirectly lead to mass loss by invoking flexure and fracture (Banwell & MacAyeal, 2015; Banwell et al., 2013; Robel & Banwell, 2019). This ponding meltwater decreases ice‐shelf stability (Scambos et al., 2000, 2009) and may allow faster flow of glaciers and ice streams to the ocean (Rydt et al., 2015; Scambos et al., 2004). Meltwater impacts on mass balance and dynamics are more poorly understood for the AIS than the GrIS, and the hydrologic processes driving mass loss are complex and poorly documented (Bell et al., 2018). Antarctic ice‐shelf instability may be exacerbated by atmospheric warming (Siegert et al., 2019) that results in a nonlinear increase in surface meltwater production, due to the positive melt‐albedo feedback (Trusel et al., 2015). Recent work indicates that meltwater production and ponding on the AIS are more extensive than previously thought (Bell et al., 2018; Kingslake et al., 2017). Large‐scale drainage networks transport meltwater across ice shelves, feeding surface melt ponds (Dell et al., 2020; Kingslake et al., 2017; Phillips, 1998) and, in some cases, exporting water to the ocean via large waterfalls (Bell et al., 2018). Additionally, a portion of meltwater is stored in buried lakes, which are located within the firn and can be buried up to a few meters below the ice surface (Lenaerts et al., 2017; MacAyeal et al., 2019; Miles et al., 2017). These shallow buried lakes (sometimes referred to as “subsurface” or “englacial” lakes; Bell et al., 2018) are usually invisible in optical satellite images, which makes it difficult to study their extent, evolution, and interaction with drainage systems.

Although Antarctic surface lake drainage events have been observed remotely (Langley et al., 2016; Leeson et al., 2020; Scambos et al., 2000, 2009), and lake drainage via stream overflow has been documented with field measurements on the McMurdo Ice Shelf (Banwell et al., 2019), until this study, there have been no field observations of vertical lake drainage on the AIS via the rapid hydrofracture mechanism.

**Figure 1:**
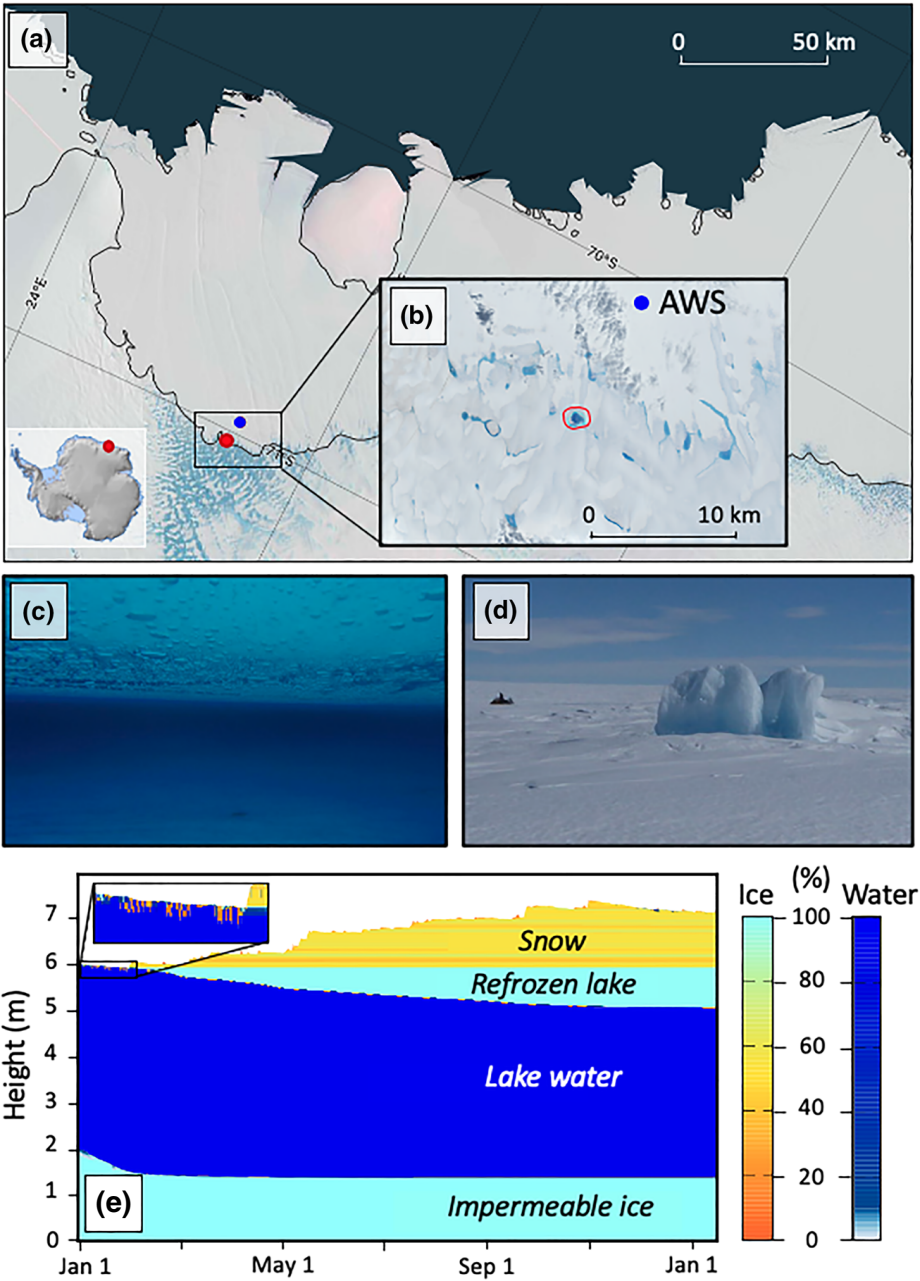
Roi Baudouin Ice Shelf (RBIS) buried lake study region. (a) Overview map of the RBIS, East Antarctica, with MODIS mosaic (Scambos et al., 2007) in the background showing patterns of blue ice near the ice‐shelf grounding line (black line, MEaSUREs grounding line definition; Rignot et al., 2016). Blue dot represents AWS location. (b) Visible Landsat 8 image (6 January 2017) of surface meltwater near the RBIS grounding line. (c) Photo from within the buried lake (photo credit: Stef Lhermitte). (d) Photo of the postcollapse ice ridges/blocks (photo credit: Stef Lhermitte). (e) Surface meltwater evolution simulated by a snow model driven by AWS data from January 2015 to February 2016.

Here, we first use a snow model to investigate the formation and subsequent evolution of a buried lake discovered by a Belgian field team in February 2016, near the grounding line (about 1 km inland from the MEASUREs grounding line definition; Rignot et al., 2016) of the Roi Baudouin Ice Shelf (RBIS; 26.30°E, 71.03°S) in Dronning Maud Land, East Antarctica (Figures 1a–1c). In December 2017, the field team returned to the site and found that the perennial buried lake had drained, causing an ice surface lowering, and the formation of a series of ice ridges and blocks on the surface (Figure 1d), evidence of a rapid drainage event (Das et al., 2008; Tedesco et al., 2013). We analyze ground penetrating radar (GPR) transects to show that the buried lake in the grounding zone of the RBIS drained via vertical fractures, providing the first direct evidence of hydrofracturing on the AIS. By comparing fracture locations from two different field campaigns, we show that these fractures, which are reactivated by meltwater, propagate onto the ice shelf, potentially providing structural weaknesses in the ice‐shelf surface. Finally, we show that a decrease in surface elevation accompanies the lake drainage event. This evidence, along with an increase in microwave backscatter, provide additional remote insight into the temporal evolution of the buried lake.

## 2 Data and Methodology

### 2.1 Buried Lake Modeling

To simulate surface meltwater evolution, we used the detailed, physics‐based, multilayer snow cover model SNOWPACK (Bartelt & Lehning, 2002; Lehning, Bartelt, Brown, Fierz, et al., 2002; Lehning, Bartelt, Brown, & Fierz, 2002). The model was forced with half‐hourly measurements of air temperature, relative humidity, wind speed, and incoming shortwave and longwave radiation from an automatic weather station (AWS) located ∼10 km from the buried lake at 26.2553°E, 70.9624°S (Figure 1a). The AWS was operational from December 2014 to February 2016. NASA's Modern‐Era Retrospective analysis for Research and Applications, Version 2 (MERRA‐2), reanalysis product (Gelaro et al., 2017) was used for 3‐hourly precipitation input. Based on a comparison between simulated surface height increase by SNOWPACK and measured snow depth at the AWS, we found that MERRA‐2 precipitation provided an accurate assessment of the annual accumulation, even though individual accumulation events were overestimated or underestimated. The SNOWPACK model calculates the subsurface energy balance for the lake. When the lake appears as surface meltwater, the surface albedo, as well as shortwave absorption in the water layers, follows Lüthje et al. (2006). Otherwise, Michlmayr et al. (2008) is used as an albedo parameterization for the snow cover surface based on snow properties. The model uses the resulting energy balance to calculate lake freezing and thawing. Precipitation is only allowed to accumulate when at least the uppermost layer of the lake is frozen.

### 2.2 Ground Penetrating Radar

To analyze the subsurface ice layers at our field site, GPR observations were acquired in a kilometer‐wide grid using a GSSI SIR‐3000 radar system with a monostatic 400 MHz antenna. The system acquired 2,048 samples per scan at a sampling interval of 0.29 ns. We obtained antenna positional information using a Trimble GPS, which was linked to the GPR via a timestamp. The GPS data were processed using rtklib software and have an uncertainty of less than ±50 mm. Acquired GPR profiles were subject to a data processing workflow that included distance normalization, which resulted in a lateral spacing of 0.25 m/scan and horizontal background noise removal, which consisted of two steps: (1) computing and subtractive removal of the average trace from each trace in the record and (2) removal of antenna‐related noise using a lateral high‐pass filter. The width of the horizontal filter was optimized to ensure that structural‐related features were not also removed. We also applied large‐window 2‐D automatic gain control (AGC) using 100 and 6 points in the vertical and horizontal directions to balance amplitudes and account for signal attenuation with depth. Finally, we performed a 2‐D poststack depth migration to focus the observed diffractions assuming relative permittivity values of *ε*_*ice*_ = 3.1 and *ε*_*water*_ = 81 within the ice and water zones, respectively.

To obtain lake depth and volume, we trace the lake‐top and lake‐bottom in precollapse GPR transects. We use the relative permittivity values above to convert from two‐way travel time to depth. Lake depth is calculated by subtracting the lake‐top depth from the lake‐bottom depth, and volume is calculated by spatially integrating lake depth.

### 2.3 Digital Elevation Models

To investigate surface elevation changes over our study lake, we obtained digital elevation models (DEMs) from the Polar Geospatial Center's Reference Elevation Model of Antarctica (REMA) at 8 m resolution (Howat et al., 2019). The DEMs are coregistered to a reference point cloud to minimize offsets using the NASA Ames Stereo Pipeline (ASP) (Moratto et al., 2010; Shean et al., 2016). Vertical uncertainties are calculated following Zheng et al. (2018), whereby the differences between the DEM and reference point cloud are identified, and the standard deviation is calculated after clipping outliers. Three DEMs were differenced to obtain surface elevation changes between 13 September 2013 and 9 April 2015, and between 9 April 2015 and 15 December 2016.

### 2.4 Satellite SAR Backscatter Anaylsis

To investigate microwave radar backscatter changes resulting from the lake collapse, we used Level‐1 Ground Range Detected SAR data from the European Space Agency's Sentinel‐1 satellite (Copernicus, 2019). Using the Alaska Satellite Facility's Vertex data portal, we compared Sentinel‐1A tiles at 20 × 40 m^2^ resolution every 12 days over the area of interest from 1 January 2016 to 1 June 2016. The incident angle over the lake was approximately 35°. We used the HH (horizontally transmitted and received) polarization, as this was the only polarization available over this area and time period. We followed the data preprocessing methodology outlined in Miles et al. (2017) by using the Sentinel Application Platform toolbox to carry out a radiometric calibration, a single product speckle filter, and a terrain correction on each raw Sentinel‐1 scene. We performed the terrain correction using the Radarsat Antarctic Mapping Project Digital Elevation Model, Version 2 (Liu et al., 2015). Finally, consecutive Sentinel‐1 tiles were differenced to obtain 12 day backscatter difference maps.

## 3 Results

### 3.1 Buried Lake Modeling

We propose that buried lake formation may result from surface meltwater produced through the wind‐albedo feedback, whereby katabatic winds from the Antarctic interior warm and mix the air as they flow downward, causing widespread snow erosion and exposing blue ice and firn with lower surface albedo, thus enhancing surface melt (Lenaerts et al., 2017). This surface meltwater collects in topographic depressions (Figure S1 in the supporting information) on the impermeable blue ice surface in the ice‐shelf grounding zone during the melt season (Figures 1a–1c). The meltwater gets buried and is insulated by subsequent snowfall, thus becoming a buried lake.

To explore the likelihood of this buried lake formation mechanism in more detail, we use the SNOWPACK snow model (Methods) to simulate the evolution of a meltwater lake from 1 January 2015 to 3 February 2016, the year immediately prior to the lake's discovery. The simulation begins with a surface lake, which remains largely unfrozen in the austral summer, except for some temperature‐ and radiation‐driven diurnal freeze/thaw cycles of the lake surface (Figure 1e). The simulation also suggests that shortwave radiation absorption at the lake bottom results in lake‐bottom ablation, thereby increasing lake depth. As winter sets in, consistent below‐freezing temperatures allow the top layer of surface water to freeze. Snow accumulation insulates the water underneath the frozen surface from the atmosphere, allowing subsurface water to remain liquid even throughout the cold winter months (mean winter temperatures are ≈ −21° C). Sensitivity analyses indicate that the amount of snow accumulation limits the depth to which water in the buried lake freezes, suggesting that wind‐driven snow deposition plays an important role in controlling buried liquid water persistence (Figure S2). Additional sensitivity analyses were performed by varying other atmospheric inputs, such as temperature and incoming longwave radiation, yielding negligible changes to the lake‐refreezing process (not shown).

### 3.2 Field Observations

**Figure 2:**
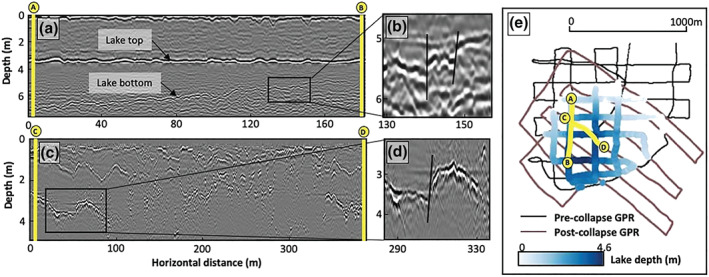
Processed GPR sections before and after the buried lake drainage event. (a) Precollapse GPR section (February 2016) showing the lake top depth (∼3.5 m) and the total water depth (≤4.6 m) along this line. (b) Evidence of near‐vertical fracturing within the lake bed. (c) Postcollapse GPR section (12 December 2017). (d) Interpreted fracture present in the ice following the lake drainage. More postcollapse fractures can be seen in Figure S3. (e) Map of precollapse and postcollapse GPR transects. Lake bathymetry is delineated by the blue gradient color scale.

GPR data were collected at the field site both before (February 2016) and after (December 2017) the buried lake drainage event. The analysis of processed precollapse GPR transects shows that the lake was buried ∼3.5 m below the ice surface, with an average water depth of 2.2 m and a maximum depth of 4.6 m (Figures 2a–2c). A linear interpolation of the GPR‐derived lake depths indicates that the lake contained ∼1.5 billion m^3^ of water. This volume is likely an underestimation because the GPR survey did not completely cover the southern‐most lake border. The GPR data also show several lake bed discontinuities, mostly in the southern portion of the lake (Figures 2b and 5a). These discontinuities suggest the presence of closed vertical fractures within the lake bed, even before its drainage, indicating that this lake may have been preconditioned to drain. These preexisting fractures were located at a horizontal distance of ∼100–300 m from the southern edge of the lake, which means they had already been present in the lake basin for >220 days prior to their discovery by the field team, assuming an ice velocity of 165 m/year in this region (Rignot et al., 2017). The preexisting fractures may be a result of previously drained meltwater ponds, and/or tidal flexure and larger‐scale ice flow stress. In the postcollapse GPR data (Figures 2d, 2e, and S3), we find several large (∼1 m) discontinuities up to 4 m below the collapsed surface and numerous smaller discontinuities in shallower ice layers 1–2 m below the ice surface. We interpret these discontinuities to be offset stratigraphy, which suggests vertical fracturing at potential lake drainage locations.

### 3.3 Remote Sensing Observations

**Figure 3:**
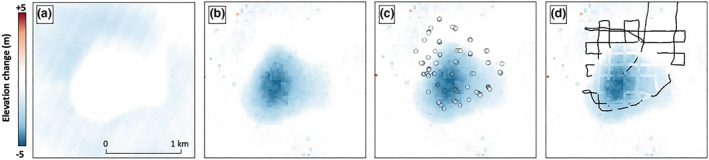
Differential DEM maps suggesting surface collapse due to buried lake drainage. (a) Predrainage elevation change between 13 September 2013 and 9 April 2015, showing little change over lake area. Elevation change between −0.5 and +0.5 m is shown in white. (b–d) Postdrainage elevation change between 9 April 2015 and 15 December 2016, showing a ∼5 m depression at the lake center. (c) Differences in field GPS measurements (small dots) from before and after the lake collapse plotted with the same color scale as the background DEM difference image. (d) Lake depth (gradient blue color scale) plotted in the same color scale as the background DEM difference image. Lake depth is calculated from GPR transects. DEMs obtained from REMA (Howat et al., 2019).

The lake drainage event is further investigated by two independent remote sensing methods. Surface elevation changes in the REMA data set (Howat et al., 2019) show little variation over the buried lake prior to collapse between 13 September 2013 and 9 April 2015, with only an ∼0.60 m ± 0.27 m increase in the southern portion of the lake (Figure 3a). We attribute this elevation increase to wind‐driven snow accumulation and/or deposition. The surface height lowering surrounding the lake area in Figure 3a can be attributed to snow redistribution and firn compaction. Between 9 April 2015 and 16 December 2016 (after the first field campaign), the REMA data set indicates an average surface height decrease of 2.56 m ± 0.53 m over the lake area, with a maximum decrease of 4.77 m ± 0.53 m (Figure 3b). This surface height decrease is consistent with differences in field GPS survey measurements from before and after the collapse (Figure 3c), as well as lake bathymetry data derived from processed precollapse GPR sections (Figure 3d), and provides remote evidence of the lake drainage event.

**Figure 4:**
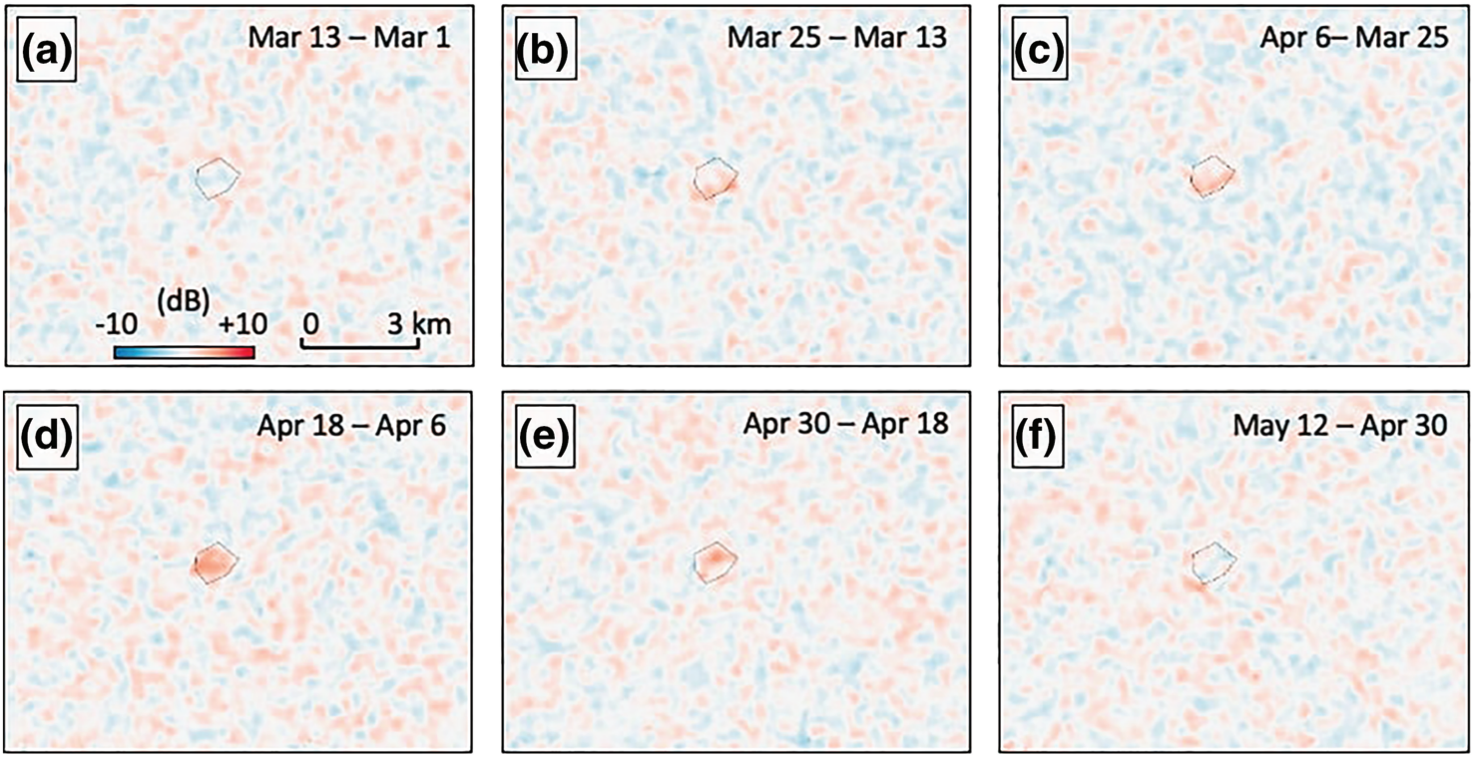
Sentinel‐1 radar backscatter differences (dB) images. Twelve‐day backscatter differences between various dates in 2016: (a) March 13 to March 1, (b) March 25 to March 13, (c) April 6 to March 25, (d) April 18 to April 6, (e) April 30 to April 18, and (f) May 12 to April 30.

**Figure 5:**
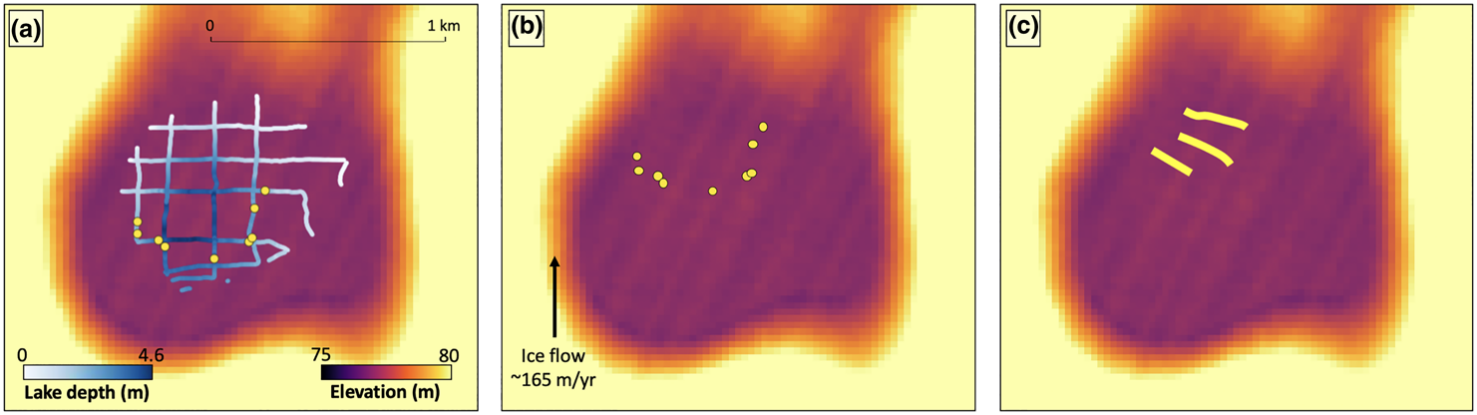
Fracture locations. (a) Location of precollapse fractures identified in GPR observations (presented in Figure 2, yellow dots) along lake bed (lake depth shown in blue gradient color scale) with REMA DEM (Howat et al., 2019) in the background. (b) Map showing where the precollapse fractures should be located at the time of the second field campaign (December 2017) assuming a MEaSURE's ice flow speed of 165 m/year^28^. (c) Map showing the actual fractured areas (yellow lines) identified in postcollapse GPR observations (yellow lines, rather than dots, are used to indicate fracture positions as there were too many postdrainage fractures to mark their individual positions).

Remotely sensed radar backscatter differences from Sentinel‐1 synthetic aperture radar (SAR) images provide additional insight into the buried lake's temporal evolution (Figure 4). Microwave radar signals are sensitive to water, even up to a few meters below the surface. Lake drainage events may lead to a radar backscatter increase as water is no longer present, and also because the surface often becomes rougher as a result of a drainage event (Miles et al., 2017). Additionally, lake freeze‐through can result in increased relative backscatter, as observed in Miles et al. (2017). The largest backscatter increase over the buried lake occurred between 25 March and 30 April 2016. One possible explanation for this relative backscatter increase is a partial freeze‐through of the buried lake, which could increase the distance from the ice surface to the lakes liquid water, such that the liquid water may then be below the penetration depth of microwave radar. This would mean that later drainage events would not be detected in the Sentinel‐1 microwave data. Alternatively, this backscatter change could represent the lake drainage event. The observed radar backscatter increase may suggest that an initial drainage occurred in the southern portion of the lake beginning 25 March 2016 (Figure 4c). This initial drainage in the southern area of the lake corresponds with the fractured area in the precollapse GPR data, supporting the hypothesis that the lake drained via these fractures (Figure S4). Subsequently, a rapid drainage event occurred over the entire lake between 6 and 18 April 2016 (Figure 4d). Finally, residual drainage occurred in the northern portion of the lake lasting until 30 April 2016 (Figure 4e). There is no lateral radar backscatter change outside of the lake basin, which may support our suggestion that the water drained vertically, not horizontally. This interpretation of backscatter signals is consistent with the presence of fractures in the southern portion of the lake bed in the precollapse GPR data (Figure 5a). In summary, we show that Sentinel‐1 radar backscatter increase over the lake area is consistent with our observations of surface elevation lowering, and may offer additional support of lake drainage during 2016. While other processes such as refreezing and ice layer formation also contribute to radar backscatter changes, none of these other processes can explain the magnitude of surface elevation change observed in the DEM data.

Landsat 8 imagery suggests that meltwater repeatedly ponds in the same topographic depression (Figures S5a–S5d). Additional Sentinel‐1 analysis from following years shows a similar increased backscatter signal (Figure S5e) during Spring 2019. This suggests that the lake may also drain recurrently; however, more field and height change observations are necessary to expand this analysis and confirm this hypothesis.

## 4 Discussion

In situ hydrological observations in Antarctica are currently limited, but we have shown in this study that they are invaluable for complementing satellite‐derived data and modeling results to examine the role of meltwater processes on the potential instability of the AIS. Field GPR measurements have shown that a buried lake drained via vertical hydrofracture near in the grounding zone of an Antarctic ice shelf. We have further exhibited that surface elevation changes from field GPS observations agree with those obtained by differencing satellite‐derived DEMs. These data have been combined with microwave radar backscatter differences to further evaluate the lake's evolution; a remote technique that expands upon a novel set of field observations.

By positively identifying evidence of hydrofracture, our field‐derived and remote sensing‐derived results, combined with knowledge that this predominantly blue ice region contains a thin low‐porosity firn layer (Lenaerts et al., 2017), provide evidence of a possible link between the supraglacial, englacial, and for further inland (and grounded) regions of Antarctica, possibly even subglacial hydrologic networks, something for which there is limited previous evidence (Bell et al., 2018). If such a link exists, it may impact subglacial dynamics and thus may have dangerous implications for ice flow acceleration (Tuckett et al., 2019). Analysis of GPR‐derived fracture locations suggests that the fractures found along the lake bed during the initial field campaign had advected from south to north, toward the ice front (Figure 5). By the time of the second field campaign, the postcollapse fractured area (Figure 5c) aligned with expectations of where the precollapse fractures would be located (Figure 5b), assuming an ice velocity of 165 m/year for this region (Rignot et al., 2017). This suggests that fractures activated by water in and above the ice shelf grounding zone could be advected onto floating ice shelves, where they could precondition an ice shelf to become unstable by providing fractures away from the grounding line for ponding surface meltwater to exploit.

The GrIS has been shown to be a self‐regulated system, whereby surface melt volume is not correlated with annual ice flow velocity, at least in marginal regions (Tedstone et al., 2015; van de Wal et al., 2015), and surface lake drainage events have less of an effect on basal lubrication and ice flow than previously assumed (Poinar et al., 2017). In contrast, Antarctic ice shelves that buttress 75% of the continent (Fürst et al., 2016) (significantly more than the floating ice around Greenland Hill et al., 2017) are vulnerable to meltwater‐induced flexure and fracture, processes that pose a direct threat to ice‐shelf stability (Banwell et al., 2013; Robel & Banwell, 2019; Scambos et al., 2000, 2009), and therefore mass loss from the AIS as a whole (Rydt et al., 2015; Scambos et al., 2004). We hypothesize that the lake observed in our study may drain recurrently, based on Landsat 8 evidence that water ponds in the same topographic depression (Figures S5a–S5d) during the melt season on an almost annual basis. While such lake drainage events are a natural phenomenon, future atmospheric warming will enhance meltwater production and ponding, in turn increasing the frequency and impact of these drainage events on AIS ice shelves.

## Supporting information



Supporting Information S1

Figure S1

Figure S2

Figure S3

Figure S4

Figure S5

## Data Availability

Weather station, GPR, and GPS data are freely available at https://zenodo.org/record/3609294#.Xh-fGxdKhTY(weather station), https://zenodo.org/record/3609292#.Xh-fKRdKhTY (pre‐collapse GPR and GPS), https://zenodo.org/record/3609290#.Xh-fQRdKhTY (postcollapse GPR and GPS) or by contacting the corresponding author. Digital elevation models can be obtained from the Polar Geospatial Center's Reference Elevation Model of Antarctica (https://www.pgc.umn.edu/data/rema/). Geospatial support for this work was provided by the Polar Geospatial Center under NSF‐OPP Awards 1043681 and 1559691. DEMs were provided by the Byrd Polar and Climate Research Center and the Polar Geospatial Center under NSF‐OPP Awards 1543501, 1810976, 1542736, 1559691, 1043681, 1541332, 0753663, 1548562, and 1238993 and NASA Award NNX10AN61G. Computer time was provided through a Blue Waters Innovation Initiative. DEMs were produced using data from DigitalGlobe, Inc. Radar backscatter images were obtained from the Alaska Satellite Facility's Vertex data portal (https://search.asf.alaska.edu/#/?flightDirs=) and MERRA‐2 data can be accessed online (at https://gmao.gsfc.nasa.gov/reanalysis/MERRA-2/data_access/).
