# Supplementary material for: Observations of Buried Lake Drainage on the Antarctic Ice Sheet
Source: Geophys Res Lett. 2020 Jul 31;47(15):e2020GL087970. doi: 10.1029/2020GL087970 (PMC7507767; doi:10.1029/2020GL087970)
Supplement: Supplementary file 1 — Supporting Information S1 [file GRL-47-e2020GL087970-s001.pdf]

Supporting Information for "Observations of subsurface lake drainage on the Antarctic Ice Sheet"

D. Dunmire[1], J. T. M. Lenaerts[1], A. F. Banwell[2,3], N. Wever[1], J. Shragge[4], S. Lhermitte[5], R. Drews[6], F. Pattyn[7], J. S. S. Hansen[8,2], I. C. Willis[3,2], J. Miller[2], E. Keenan[1]

[1]Department of Atmospheric and Oceanic Sciences, University of Colorado Boulder, Boulder, CO, USA

[2]Cooperative Institute for Research in Environmental Sciences (CIRES), Boulder, CO, USA

[3]Scott Polar Research Institute (SPRI), University of Cambridge, Cambridge, UK

[4]Geophysics Department, Colorado School of Mines, Golden, CO, USA

[5]Department of Geoscience and Remote Sensing, Delft University of Technology, 2600 GA Delft, The Netherlands

[6]Department of Geology and Geodynamics, Universität Tübingen, Tübingen, Germany

[7]Laboratoire de Glaciologie, Université Libre de Bruxelles, Brussels, Belgium

[8]Department of Geological Sciences, University of Colorado Boulder, Boulder, CO, USA

Contents of this file

1. Figures S1 to S5

Introduction Here we provide supporting figures that help aid the understanding of this paper. Figure S1 shows how buried lakes form in topographic lows. Snow modeling

sensitivity analysis to varying amounts of snow accumulation is shown in Figure S2. Figure S3 shows additional GPR observations of post-collapse fractures. Figure S4 highlights that initial backscatter changes over the lake occurred in the same region where pre-existing fractures were located. Figure S5 provides evidence from Landsat 8 imagery that water ponds in the same topographic depression during the melt season on an almost annual basis and includes a plot which shows the temporal evolution of microwave backscatter changes over the lake from 2016-2019.

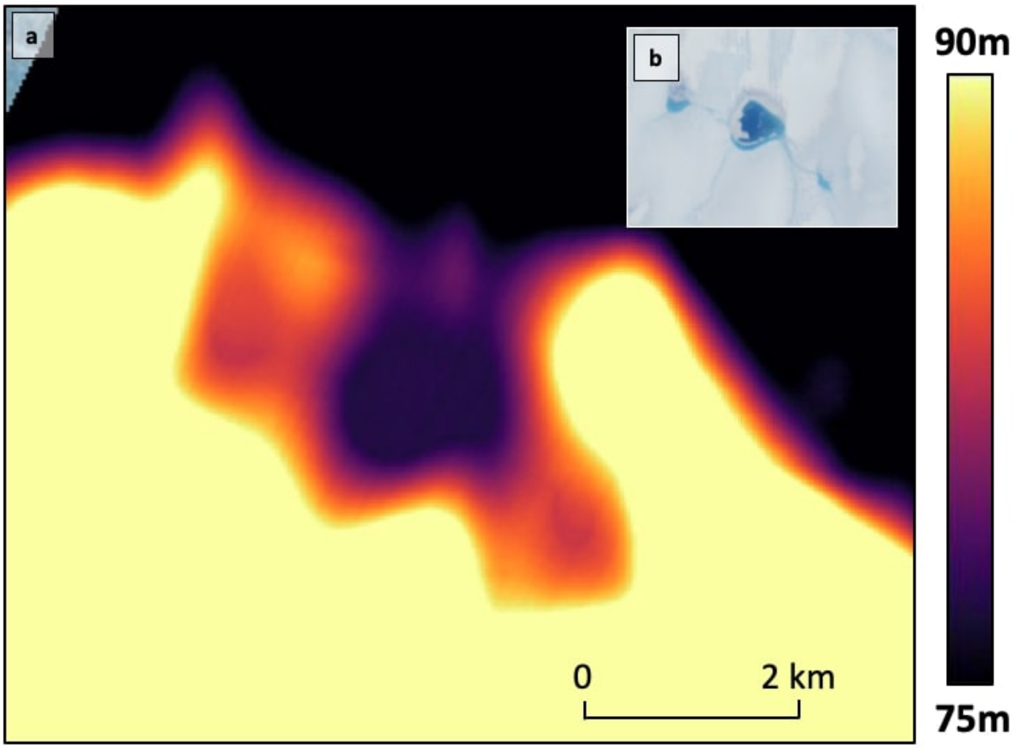

Figure S1. **a.** Elevation data of the buried lake area from REMA (Howat et al., 2019) showing how these lakes initially form in surface topographic depressions. **b** Landsat 8 image (January 6, 2017) of the same region shown in **a.** DEM obtained from REMA.

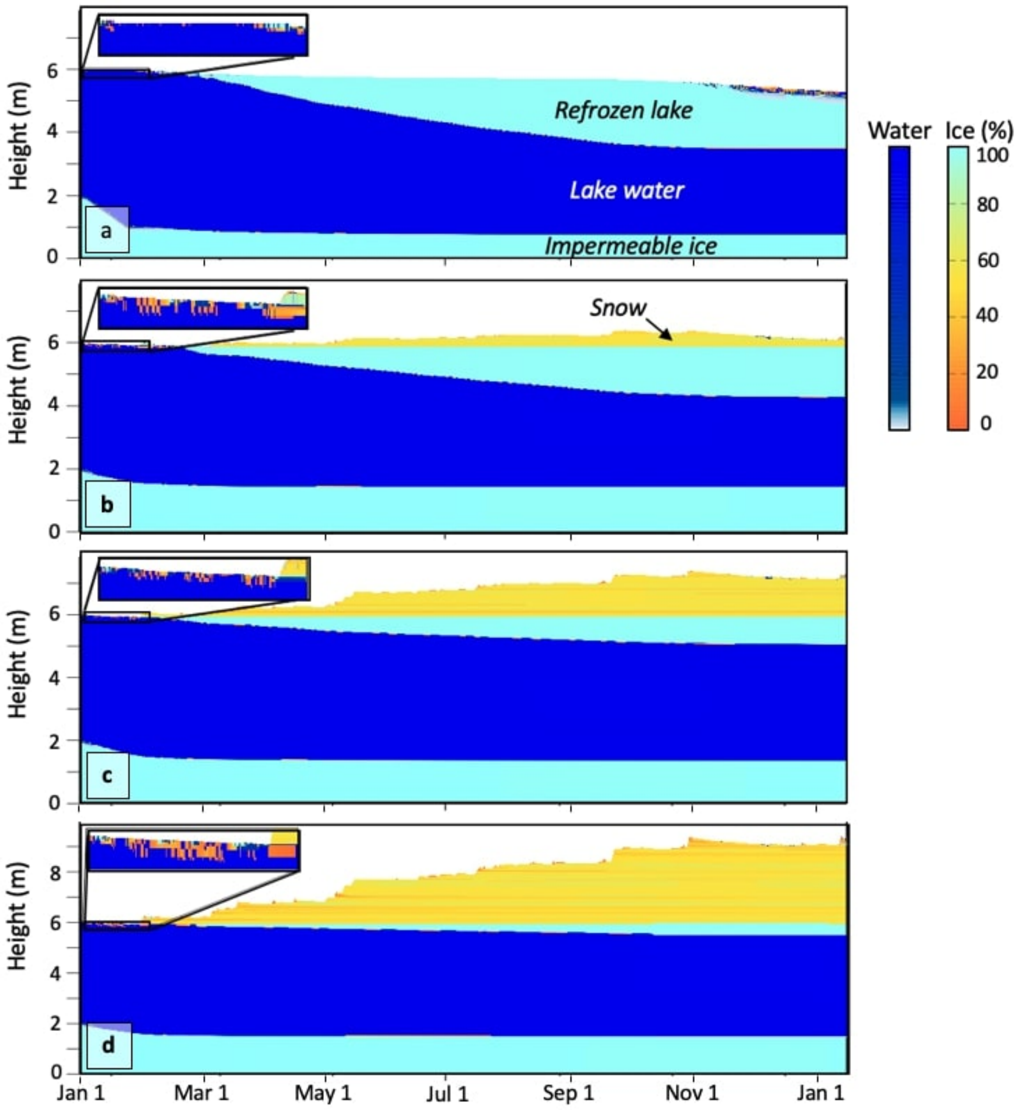

Figure S2. Results from SNOWPACK snow accumulation sensitivity analysis. SNOWPACK model runs with: **a.** no snow accumulation, **b.** half of snow accumulation from MERRA-2 (Gelaro et al., 2017), **c.** snow accumulation from MERRA-2, **d.** double snow accumulation from MERRA-2, showing how the amount of snow accumulation influences the amount of water in the buried lake that freezes.

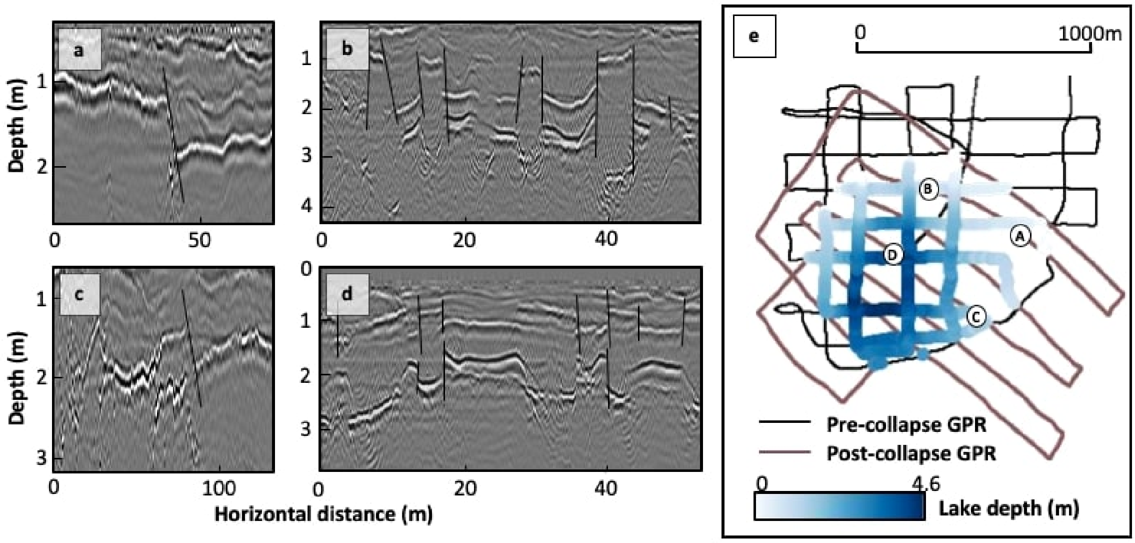

Figure S3. Fracture locations from post-collapse GPR data. **a-d.** Images of interpreted fractures from processed post-collapse GPR data. **e.** Map showing fracture locations with the former lake outlined in the gradient blue colour scale.

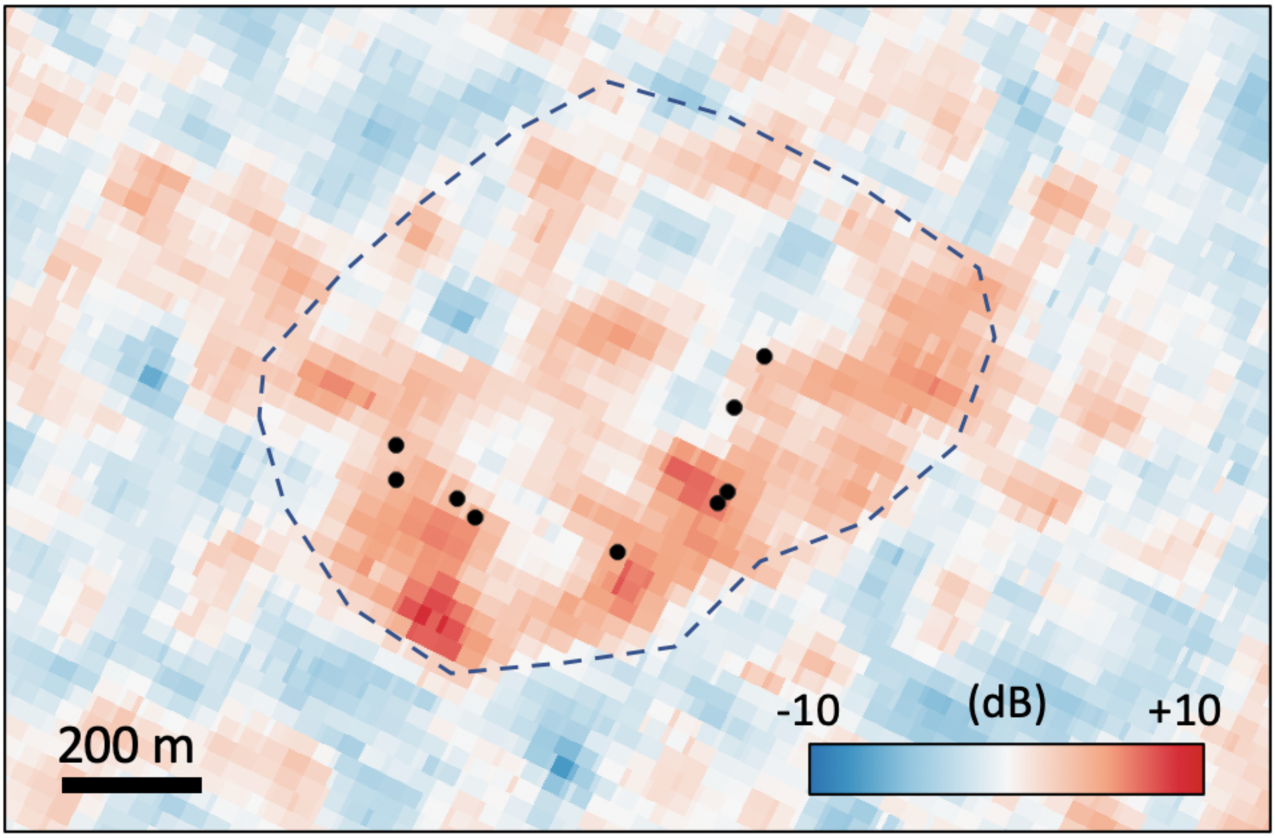

Figure S4. Pre-existing fracture locations (black dots) along the buried lake bed, detected in pre-collapse GPR transects. The background image shows Sentinel-1 backscatter change between March 25, 2016 and April 6, 2016, highlighting that initial backscatter changes occured over the same region where pre-existing fractures were located. The approximate lake borders are outlined in the dashed line, which delineates the entire area where a backscatter increase was observed.

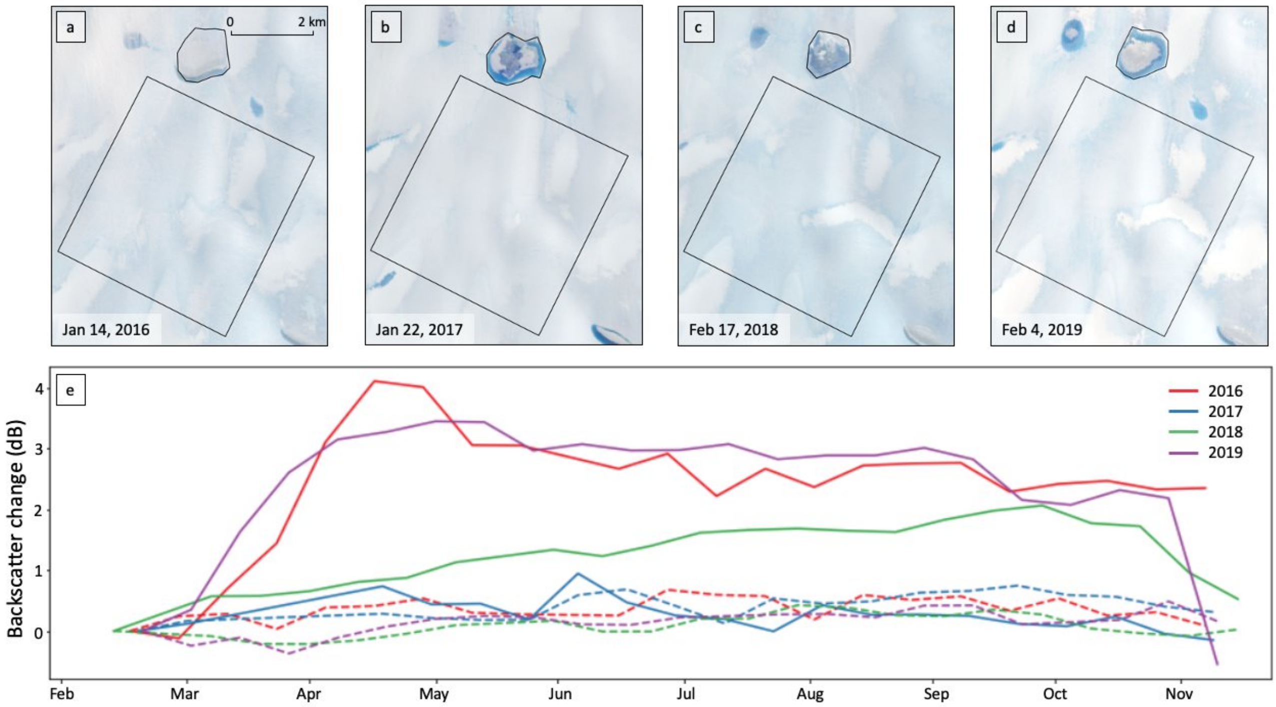

Figure S5. Evidence of recurrent lake formation and possible drainage. **a-d.** Landsat 8 optical imagery from January 14, 2016 (**a**) January 22, 2017 (**b**), February 17, 2018 (**c**), and February 4, 2019 (**d**). The blue coloration in the southern portion of the lake in **a** is exposed blue ice. **e.** Sentinel-1 backscatter changes (normalized to start at 0) from February 16 - November 31 for 2016 (red), 2017 (blue), 2018 (green), and 2019 (purple) over the study lake (solid) and background area (dashed). Lake and background area are delineated in black in **a-d.**

References

Gelaro, R., McCarty, W., Suárez, M. J., Todling, R., Molod, A., Takacs, L., et al. (2017). The modern-era retrospective analysis for research and applications, version 2 (merra-2). *AMS*. doi: https://doi.org/10.1175/JCLI-D-16-0758.1

Howat, I. M., Porter, C., Smith, B. E., Noh, M. J., & Morin, P. (2019). The Reference Elevation Model of Antarctica. *The Cryosphere*, *13*, 665-674. doi: https://doi.org/10.5194/tc-13-665-2019
