# Supplementary figures and images for: Observations of Buried Lake Drainage on the Antarctic Ice Sheet
Source: Geophys Res Lett. 2020 Jul 31;47(15):e2020GL087970. doi: 10.1029/2020GL087970 (PMC7507767; doi:10.1029/2020GL087970)

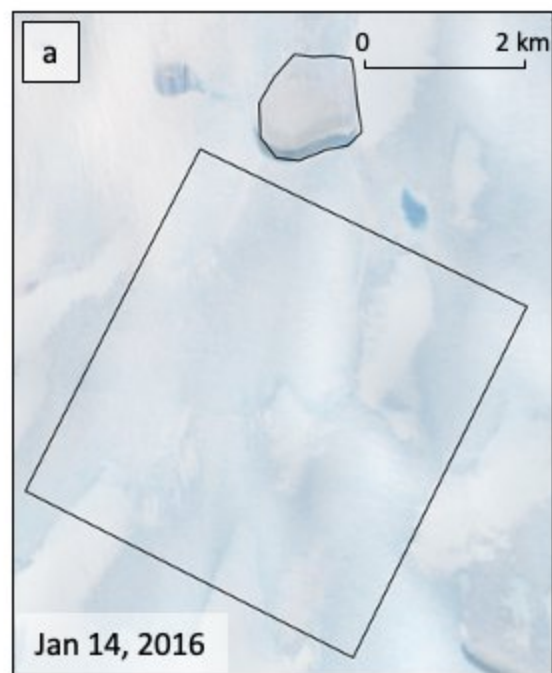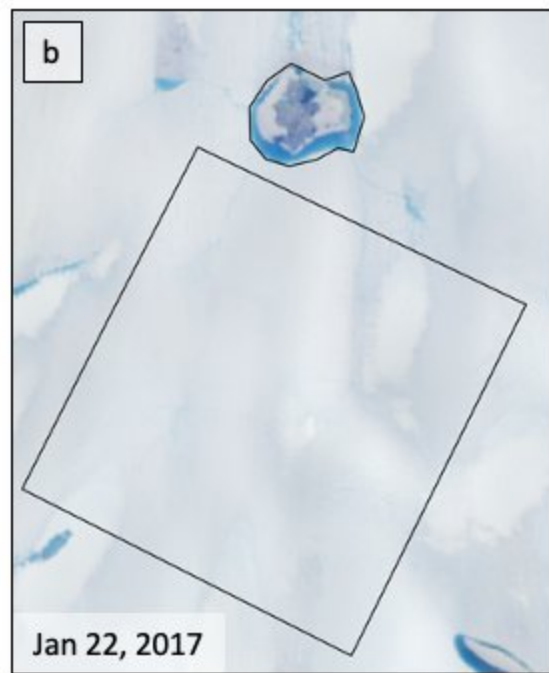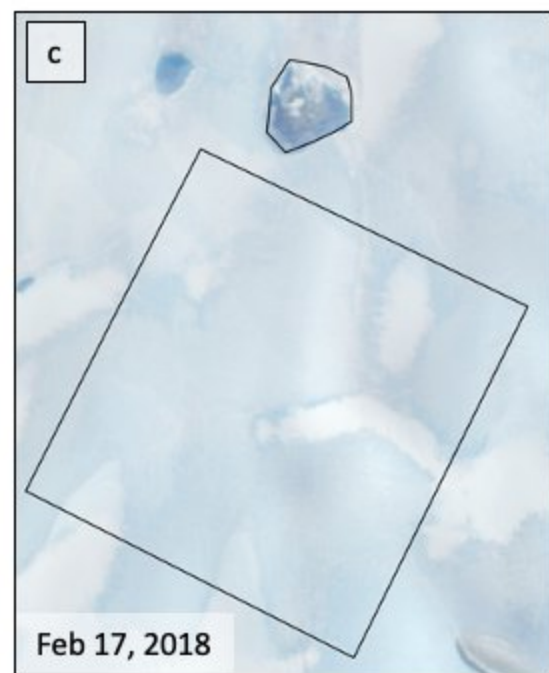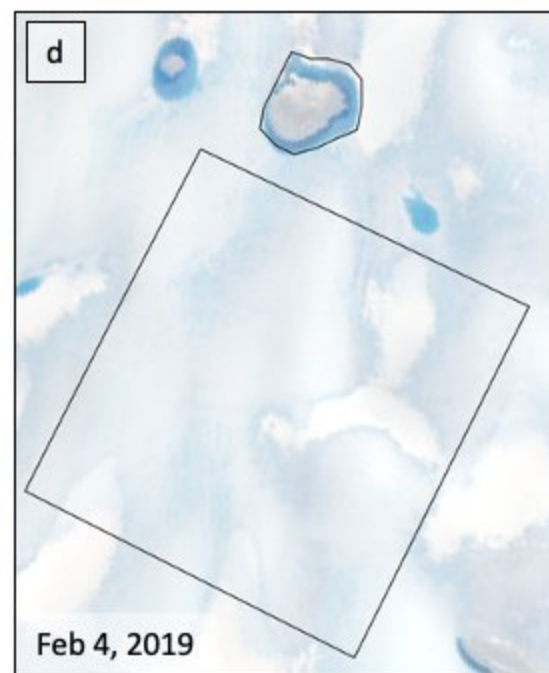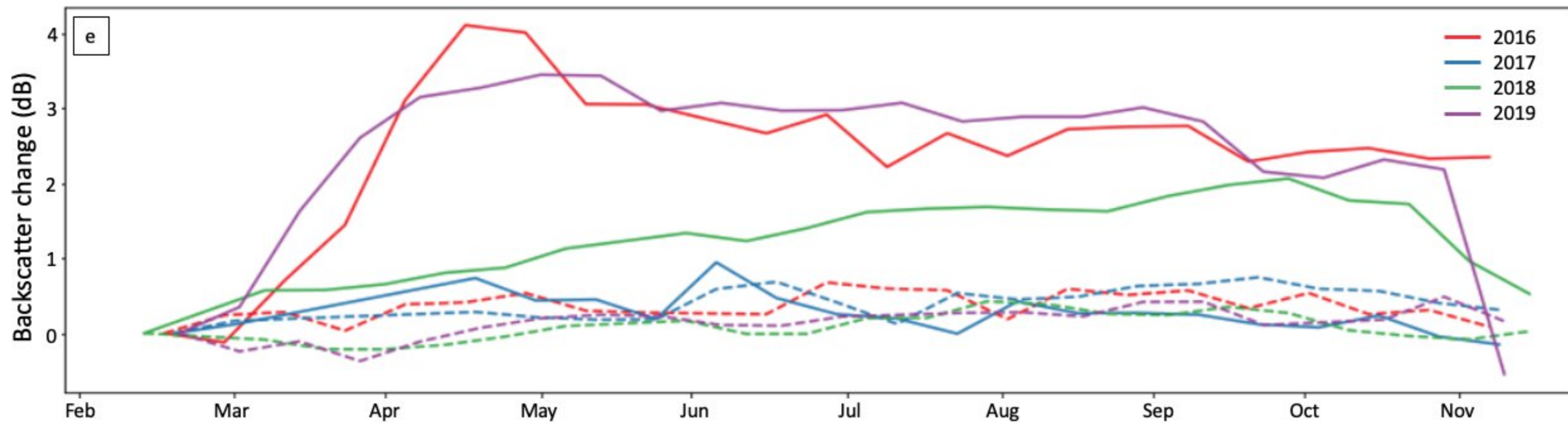

Supplement: Supplementary file 2 — Figure S1 [file GRL-47-e2020GL087970-s002.pdf]

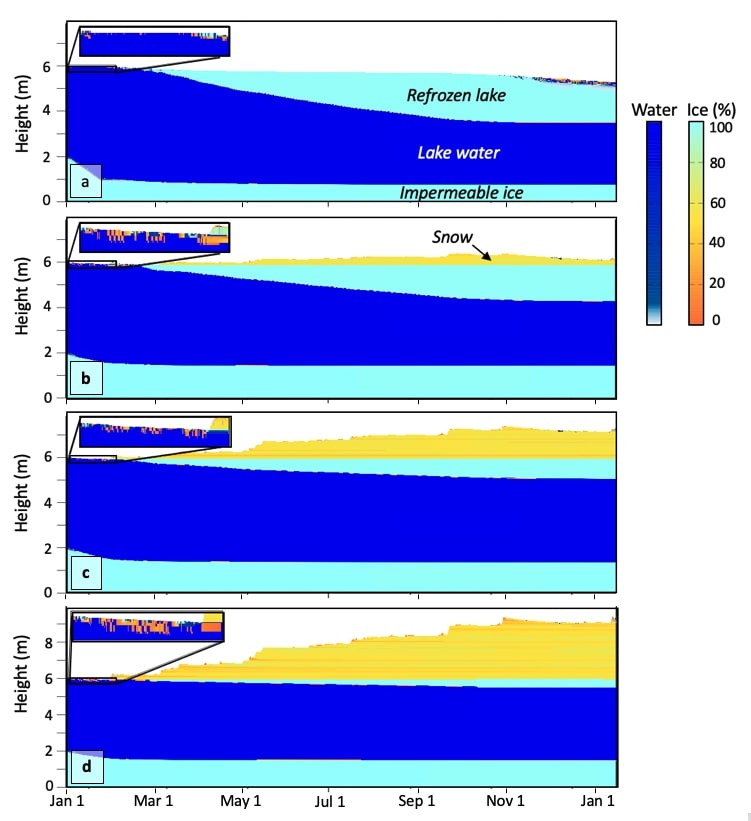

Supplement: Supplementary file 3 — Figure S2 [file GRL-47-e2020GL087970-s003.jpg]

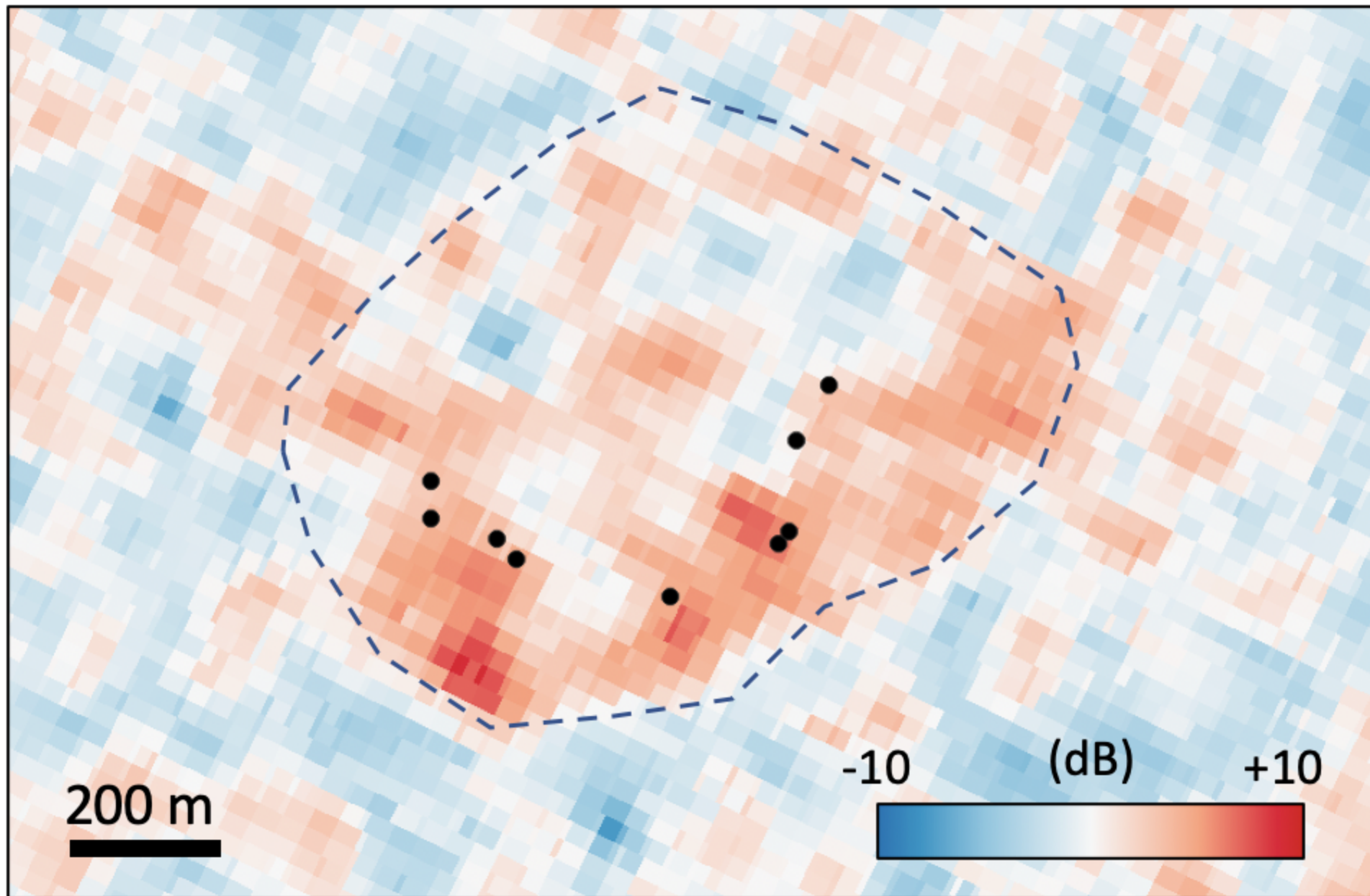

Supplement: Supplementary file 4 — Figure S3 [file GRL-47-e2020GL087970-s004.pdf]

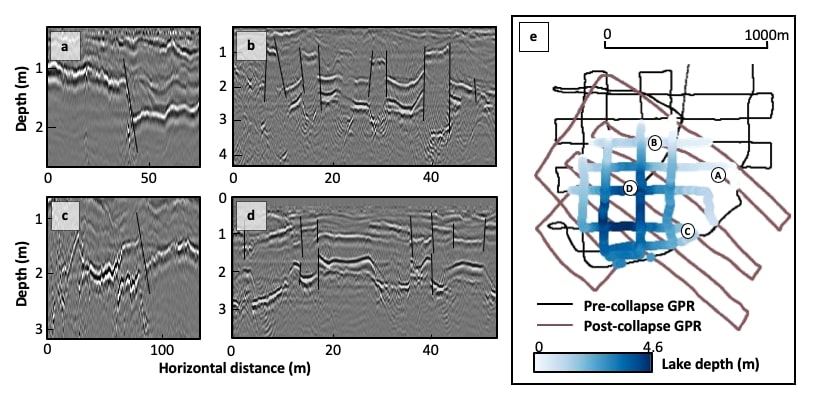

Supplement: Supplementary file 5 — Figure S4 [file GRL-47-e2020GL087970-s005.jpg]

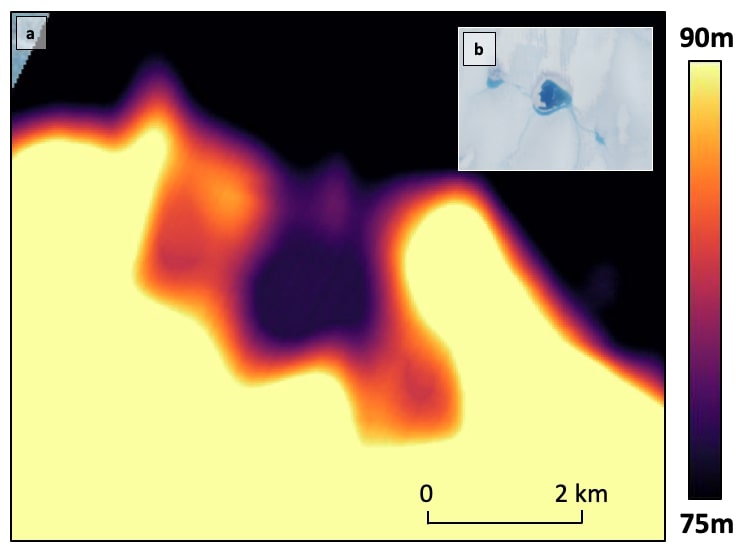

Supplement: Supplementary file 6 — Figure S5 [file GRL-47-e2020GL087970-s006.jpg]
